# Length–Weight Relationship, Age, and Growth of Invasive *Carassius auratus* in Lugu Lake, China

**DOI:** 10.3390/ani15081091

**Published:** 2025-04-09

**Authors:** Kaifei Li, Jinling Gong, Feifei Hu, Zhibin Guo, Zhaoyuan Lu, Mingzhong Luo, Tingbing Zhu

**Affiliations:** 1 College of Animal Science and Technology, Yangtze University, Jingzhou 434020, China; 2 Key Laboratory of Freshwater Biodiversity Conservation, Ministry of Agriculture and Rural Affairs, Yangtze River Fisheries Research Institute, Chinese Academy of Fishery Sciences, Wuhan 430223, China

**Keywords:** invasive species, population structure, von Bertalanffy model, plateau lake

## Abstract

*Carassius auratus*, a key invasive species in the high-altitude Lugu Lake (2690 m) in China, was studied to assess its length–weight relationship, age structure, and growth patterns using scale-based age determination. A total of 670 specimens were collected via systematic gillnet surveys conducted in May and October 2023. The population comprised five age groups (0+ to 4+), with 0+ and 1+ individuals dominating (69.40%). Body lengths ranged from 36 to 178 mm (mean: 93.73 mm), peaking at 60–70 mm (33.88%). Body weights varied from 1.3 to 175 g (mean: 33.60 g), with 1.3–10 g as the dominant range (26.27%). The length–weight relationship was modeled as *W* = 2 × 10^−5^*L*^3.026^ (*n* = 670, *R*^2^ = 0.985), indicating isometric growth. Von Bertalanffy growth parameters indicated an asymptotic length (*L*∞) of 401.57 mm, an asymptotic weight (*W*∞) of 484.38 g, a growth coefficient (*k*) of 0.0721, and a theoretical age at zero length (*t*_0_) of −1.6174 years, with an inflection point at 6.66 years. The simplified age structure and dominance of young cohorts suggest rapid population expansion, highlighting the urgent need for management strategies to mitigate ecological impacts on this fragile plateau ecosystem.

## Simple Summary

Lugu Lake, a high-altitude lake located in Southwest China, is currently threatened by invasive fish species. *Carassius auratus* is the most dominant invasive fish species in Lugu Lake; however, there are no existing reports on its population characteristics. This study presents an analysis of the population structure and growth characteristics of *C. auratus* in Lugu Lake, utilizing scale-based age determination and growth modeling. The findings indicate that the *C. auratus* population in Lugu Lake is primarily composed of young individuals and is experiencing rapid growth. To mitigate the further proliferation of the *C. auratus* population, it is recommended that management authorities implement measures such as centralized fishing to remove *C. auratus*, enforce strict controls on the release of invasive fish, and enhance artificial breeding and stock enhancement of native fish species.

## 1. Introduction

Lugu Lake is situated at the border of Sichuan Province in the southwest and Yunnan Province in the northwest. Geographically, Lugu Lake is part of the Jinsha River system. Lugu Lake is a deep-water plateau lake, situated at an altitude of 2690 m. The portion of the lake with a depth greater than 50 m accounts for approximately half of its total lake surface area, which has an average depth of 40.3 m. The lake has a water volume of 2.252 billion cubic meters [1]. It is slightly oriented in a northwest-southeast direction, measuring 9.5 km in length from north to south and 5.2 km in width from east to west. The total surface area of the lake is approximately 50.1 km^2^ [1]. According to a comprehensive analysis of the survey data, there are 15 species of fish in Lugu Lake, belonging to 5 orders and 6 families. Among these, there are four indigenous species, including *Misgurnus anguillicaudatus* (Cantor, 1842), *Schizothorax labrosus* (Wang, Zhuang & Gao, 1979), *Schizothorax ninglangensis* (Wang, Zhuang & Gao, 1979), and *Schizothorax microstomus* (Hang, 1985) [2]. The remaining 11 species are alien species that are common species widely distributed in the main stream and tributaries of the Yangtze River. Among these, *Cyprinus carpio* (Linnaeus, 1758), *Carassius auratus* (Linnaeus, 1758), *Ctenopharyngodon idella* (Valenciennes, 1844), and *Protosalanx hyalocranius* (Abbott, 1901) are the primary economic fish in Lugu Lake [3]. As alien species have gradually infiltrated Lugu Lake, the fish fauna in the lake have become dominated by alien species, leading to the endangerment of three endemic *Schizothorax* fish species [2,4].

*C. auratus* is a species of economic importance within the Cyprinidae family, naturally found in freshwater environments across China, with the exception of plateau regions. Due to its tender flesh, delightful flavor, and high nutritional value, it has become a traditional aquatic delicacy favored by many. However, due to its strong adaptability to various environments, early sexual maturity, and rapid reproductive cycles, it contributes to its potential for population overgrowth [5,6,7]. In recent years, this species has invaded the main stream of the Yarlung Zangbo River [8,9,10], Lhasa River [11], Niyang River [12,13], Lalu Wetland [14], and Chabalang Wetland [15,16], establishing itself as a dominant species in these plateau ecosystems.

Currently, there are relatively few reports on fish in Lugu Lake. Peng et al. [3] and Kong et al. [17] employed traditional survey methods to investigate fish resources in Lugu Lake, primarily focusing on fish composition, fauna, and biological habits. However, there remains a significant gap in more in-depth research regarding the age structure of fish populations, which hampers effective fisheries resource management. This study aims to analyze the age structure, growth characteristics, and length–weight relationship (LWR) of the invasive Carassius auratus population in Lugu Lake, China, using scale-based age determination. The LWR serves as a key indicator of fish population dynamics, offering insights into resource allocation strategies and adaptability under plateau-specific ecological pressures. Combined with von Bertalanffy’s growth modeling, these analyses collectively reveal the life history traits that underpin the species invasive success. The aim is to understand the age and growth characteristics of the invasive *C. auratus* in Lugu Lake, providing a scientific basis for controlling its population and supporting ecological restoration of the lake’s threatened native fish communities. These findings will directly inform strategies for sustainable management, balancing invasive species mitigation with the conservation of endemic biodiversity.

## 2. Materials and Methods

### 2.1. Sampling Point Settings

**Figure 1:**
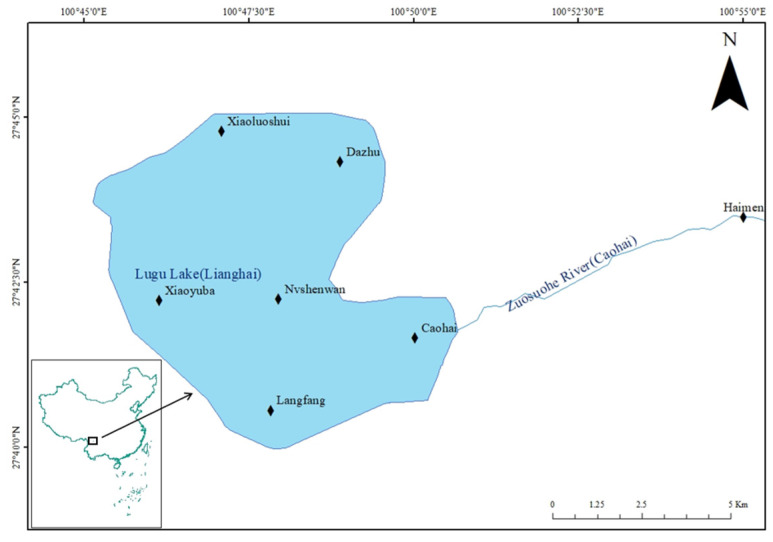
Map of sampling sites of *C. auratus* in Lugu Lake.

According to the shape and hydrological characteristics of Lugu Lake, a total of seven sampling points were set up in the lake area and its outflow tributaries: Dazhu, Xiaoluoshui, Xiaoyuba, Nvshen Bay, Langfang, Caohai, and Haimen (Figure 1). The key environmental parameters of each sampling point are shown in Table 1, and the values are the means measured in May and October 2023.

### 2.2. Sample Collection and Processing

Fish samples were collected in May and October 2023. At each sampling point, fish were captured using two pairs of floating nets (100 m long, 8 m high, 6 cm mesh), three pairs of sinking nets (100 m long, 1.5 m high, with mesh sizes of 2 cm and 7 cm), and three trap cages (10 m long, 0.45 m wide, 0.33 m high, 0.4 cm mesh). The nets were deployed in the open water at the sampling site in the evening and retrieved the following morning. For the collected specimens of *C. auratus*, body length was measured using a ruler (measuring range: 50 cm, precision: 1 mm), and body weight was determined with an electronic balance (measuring range: 3 kg, precision: 0.1 g). Additionally, 5–10 scales were collected from each fish, located between the anterior and inferior dorsal fins and along the lateral line, and were collected from each tail and brought back to the laboratory for age determination [18].

After soaking the scales in a 2% NaOH solution for 24 h, five well-preserved scales were selected for sealing. Following drying, the scales with a regular shape and clear ring lines were examined under an anatomical microscope for age identification, as well as for measuring annulus radius and scale length [19]. The age was determined and counted according to the method described by Deng et al. [20], where the annulus radius (*r_n_*) refers to the distance from the scaly core to the annulus on the positive side, while the scale radius (*R*) is the distance from the scaly core to the edge of the positive side. Age groups were recorded as 0+, 1+, 2+, and so forth, corresponding to ages 0, 1, and 2, respectively. Finally, the second round of identification was conducted two weeks after the initial age assessment, followed by a third round, and the results were averaged.

### 2.3. Data Analysis

#### 2.3.1. Length–Weight Relationships (LWRs)

The Keys formula is utilized to model the relationship between body length and body mass, as presented in the following formula [21,22]:

*W* = *a* × *L^b^*,

where *L* represents body length (mm), *W* represents body mass (g), *a* is a constant, and *b* is a power exponent. A t-test was conducted to analyze whether there was a significant difference between the *b* value and 3 in order to determine whether the fish population exhibited an isometric growth pattern [9].

#### 2.3.2. Relationship Between Body Length and Scale Radius

The relationship between body length and scale radius was fitted by the Rosa Lee equation, and the body length of the sample was calculated as follows:

*L* = *a* + *bR*,

where *L* represents the body length (mm), *R* represents the scale radius (mm), *a* is a constant, and *b* is a power exponent.

#### 2.3.3. Growth Equation

The growth characteristics of *C. auratus* were described by the von Bertalanffy growth equation for both body length and body weight.

Body length growth equation: *L_t_ = L*_∞_[1 − e^–*k*(*t − t*0)^]

Body weight growth equation: *W_t_ = W*_∞_[1 − e^–*k*(*t − t*0)^]*^b^*

*L_t_* and *W_t_* represent the body length (mm) and body mass (g) at age *t*, respectively. *L*_∞_ and *W*_∞_ are the asymptotic body length and asymptotic body mass, respectively. *t_0_* is the assumed theoretical starting age for growth, *k* is the growth coefficient, and b is the power index in the relationship between body length and body weight [23,24].

#### 2.3.4. Growth Rate, Acceleration, and Growth Inflection Point

The growth rate equation and the growth acceleration equation are derived by solving the first-order and second-order derivatives of the growth equation. The parameters *L*_∞_, *t_0_*, and *k* of the growth equation are estimated using the least square method [25]. The age at which the growth inflection point occurs is determined by d^2^*W*/d*t*^2^ = 0, that is, *t_i_* = ln*b*/*k* + *t_0_*. The statistical analysis of the data was performed using Microsoft Excel 2013.

## 3. Results

### 3.1. Body Size and Age Structure

**Figure 2:**
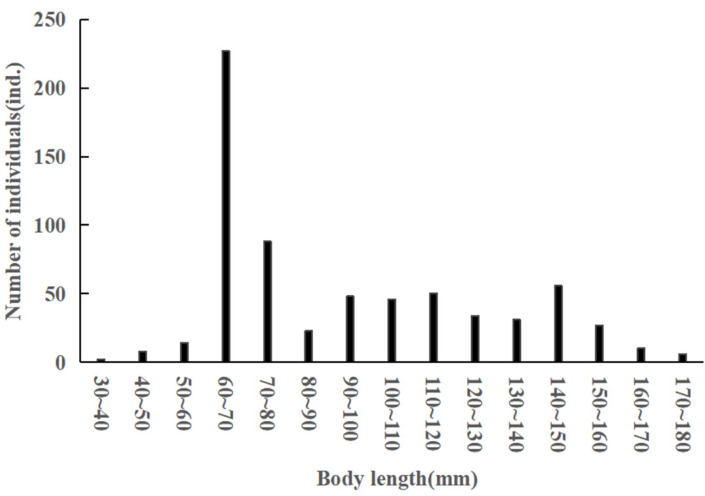
Body length distribution of *C. auratus* in Lugu Lake.

**Figure 3:**
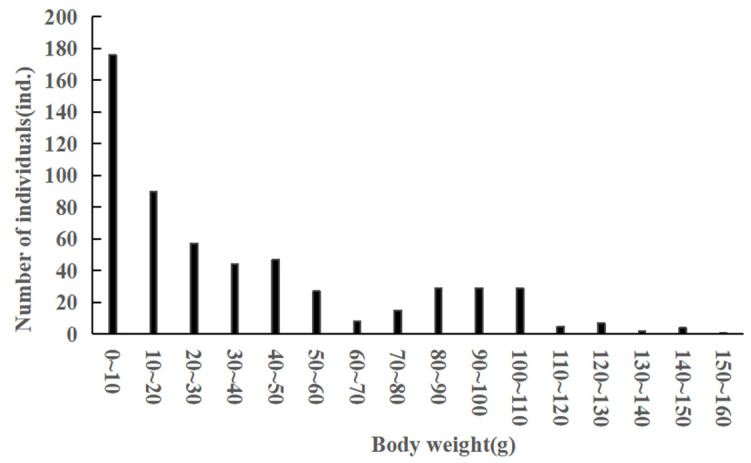
Body weight distribution of *C. auratus* in Lugu Lake.

A total of 670 *C. auratus* samples were collected. The body length ranged from 36 to 178 mm, with an average body length of 93.7 ± 32 mm. The dominant body length was 60–70 mm, accounting for 33.88% (Figure 2). The body weight varied from 1.3 to 175 g, with an average body weight of 33.60 ± 34.81 g. The dominant body weight was between 1.3 and 10 g, representing 26.27% (Figure 3).

**Figure 4:**
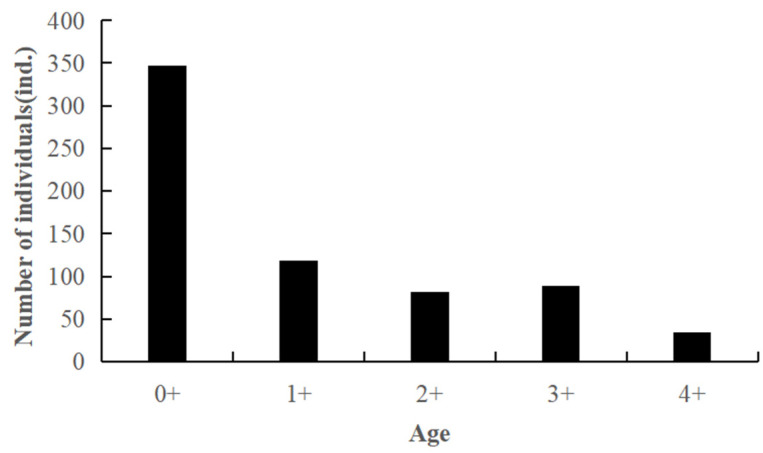
Age distribution of *C. auratus* in Lugu Lake.

The age distribution of *C. auratus* in Lugu Lake encompasses five age groups: 0+, 1+, 2+, 3+, and 4+ years. Among these, the 0+ and 1+ age groups constitute the majority, accounting for 69.40% of the total population. In contrast, the number of individuals aged 2+, 3+, and 4+ years is relatively small (Figure 4). This indicates that the age composition of *C. auratus* in Lugu Lake is simplistic, with a significant proportion of young individuals.

### 3.2. Length–Weight Relationships (LWRs) and Scale Radius Relationships

**Figure 5:**
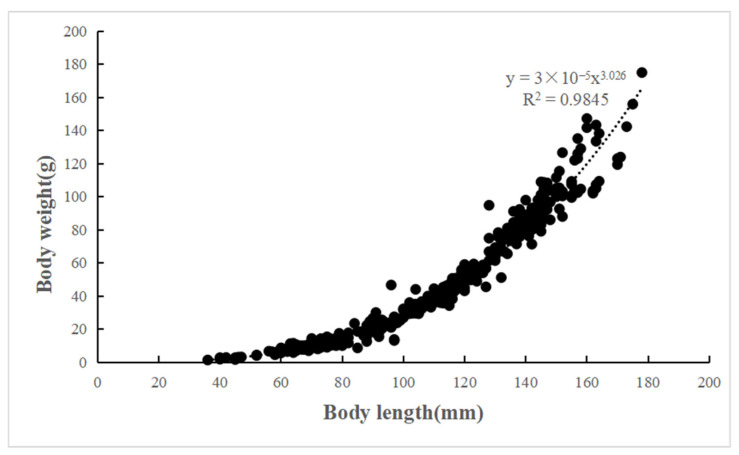
Relationship between body length and body weight of *C. auratus* in Lugu Lake.

**Figure 6:**
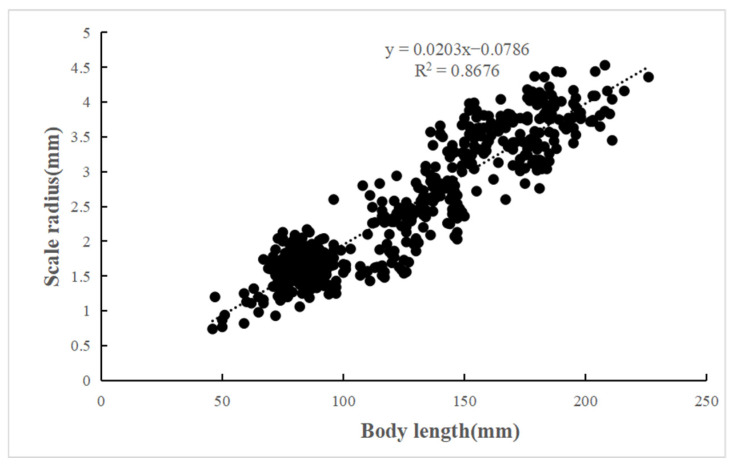
Relationship between body length and scale radius of *C. auratus* in Lugu Lake.

The length–weight relationships (LWRs) of *C. auratus* in Lugu Lake are described by the equation *W* = 2 × 10^−5^*L*^3.026^ (n = 670, *R*^2^ = 0.985) (Figure 5). The t-test showed that *C. auratus* in Lugu Lake exhibits an isometric growth (since *b* = 3.026, *p* > 0.05, *t*-test confirmed no significant deviation from *b* = 3). Additionally, a significant linear relationship was found between body length and scale radius, represented by the equation *R* = 0.0203*L* − 0.0786 (n = 670, *R*^2^ = 0.868) (Figure 6).

### 3.3. Back-Calculated Body Length

The radius of each annulus on the scale was incorporated into the established relationship between body length and scale radius so as to derive the back-calculated body length for each age group (Table 2). The results of the *t*-test indicated no statistically significant difference between the calculated body length and the measured body length across all age groups (*p* > 0.05), suggesting that the calculated body length possesses a high degree of reliability [26,27].

### 3.4. Growth Equation and Growth Parameters

*C. auratus* in Lugu Lake showed isometric growth characteristics according to the relationship between body length and body weight, which can be fitted by the von Bertalanffy growth equation. Utilizing the least squares method, the parameters of the growth equation for *C. auratus* were derived from the regression analysis of body length. The determined parameters are as follows: *L_∞_* = 401.57 mm, *k* = 0.0721, *t_0_* = −1.6174, and *W_∞_* = 484.38 g [28]. By substituting these parameters into the von Bertalanffy equation, the growth equations for body length and body weight of *C. auratus* were formulated as follows: *L_t_* = 401.57(1 − e^−0.1291(*t*+1.6174)^) and *W_t_* = 484.38(1 − e^−0.1291(*t*+1.6174)^)^2.9113^.

**Figure 7:**
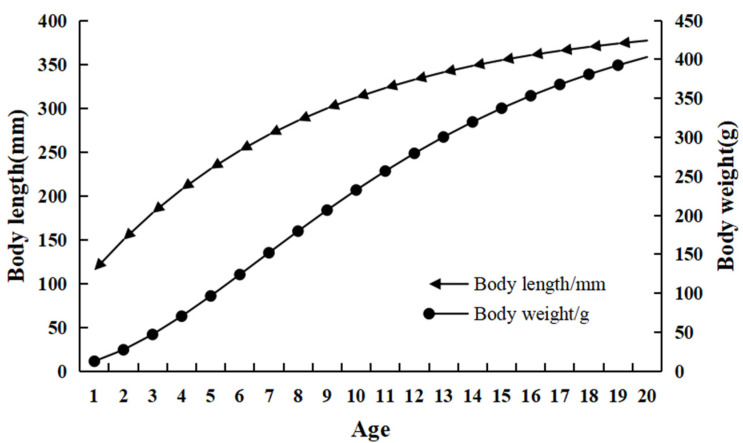
Growth curves of body length and body weight of *C. auratus* from Lugu Lake.

As shown in Figure 7, the growth curve of body length for *C. auratus* in Lugu Lake does not exhibit a distinct inflection point, instead demonstrating a gradual approach towards the asymptotic body length. Conversely, the growth curve for body mass displays an asymmetric ‘S’ shape. Initially, with advancing age, there is an acceleration in growth rate, which subsequently diminishes, ultimately approaching the asymptotic weight.

The growth rate and growth acceleration equations of *C. auratus* body length were d*_L_*/d*_t_* = 51.84e^−0.1291(*t*+1.6174)^ and d^2^*_L_*/d*_t_*^2^ = −6.69e^−0.1291(*t*+1.6174)^, respectively. The growth rate and growth acceleration equations of body weight were d*_W_*/d*_t_* = 182.05e^−0.1291(*t*+1.6174)^(1 − e^−0.1291(*t*+1.6174)^)^1.9113^ and d^2^*_W_*/d*_t_*^2^ = 23.50e^−0.1291(*t*+1.6174)^(1 − e^−0.1291(*t*+1.6174)^)^0.9113^(2.9113e^−0.1291(*t*+1.6174)^ − 1), respectively.

**Figure 8:**
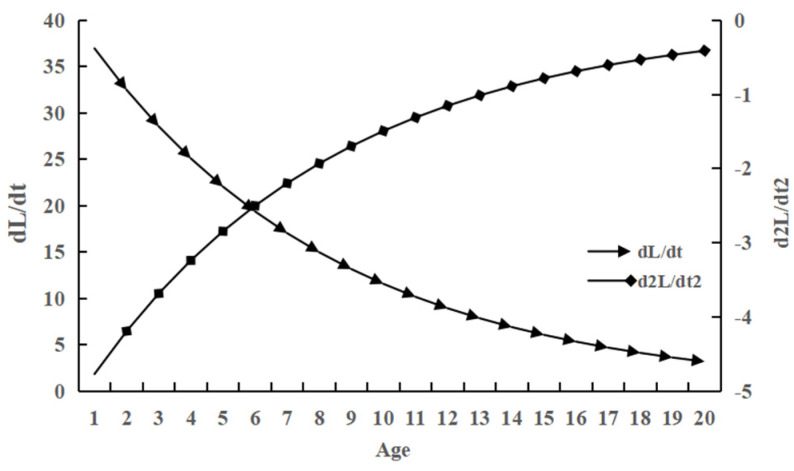
The growth rate and growth acceleration curves of body length of *C. auratus* in Lugu Lake.

**Figure 9:**
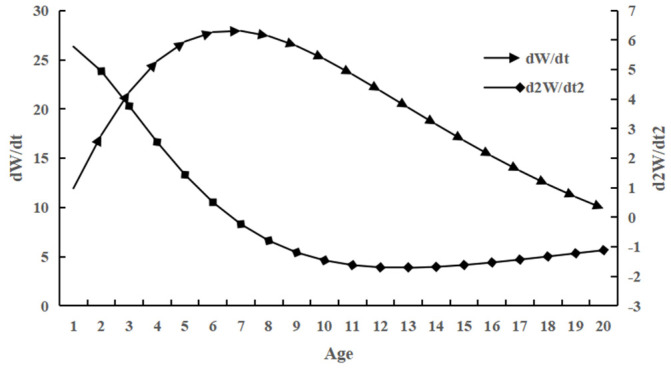
The growth rate and growth acceleration curves of body weight of *C. auratus* in Lugu Lake.

There was no inflection point in the growth rate or acceleration of body length in *C. auratus*; instead, the growth rate decreased with age and gradually approached zero. The growth acceleration consistently increased but remained negative, indicating that the decline in body length growth rate was relatively gentle (Figure 8). The curve of body weight growth rate initially increased before subsequently decreasing. Significant inflection points were observed in both body weight growth rate and acceleration curves (Figure 9). The body weight growth curve exhibited an asymmetric ‘S’ shape. Within a specific range, it demonstrated a transition from slow to rapid growth, followed by a return to slow growth as it approached the asymptote with increasing age.

## 4. Discussion

### 4.1. Age Structure of C. auratus in Lugu Lake

Fish age identification methods primarily include the hard tissue identification method and length analysis, with the hard tissue identification method being the most commonly employed [29]. The hard tissues utilized for fish age determination include scales, otoliths, vertebrae, opercula, and fins. Scales were first used for fish age identification in 1898 [17]. In this study, scales were selected as the primary material for age determination due to their non-lethal collection process, which aligns with ethical guidelines for minimizing harm to fish in Lugu Lake. While otolith analysis is widely recognized for higher accuracy, its application would require euthanizing specimens. Additionally, scales are logistically simpler to collect and process under field conditions, especially given the small body size of most individuals. However, there are limitations to using scales for age identification in *C. auratus*. For instance, in older *C. auratus*, scales may exhibit negative allometric growth or cease growing altogether. Additionally, scales may shed or wear down, resulting in blurred edges and the formation of unidentifiable structures such as secondary annuli, reproductive annuli, and regeneration annuli, which can lead to an underestimation of the sample’s age. To mitigate these biases, we implemented rigorous validation protocols: multiple scales per individual were analyzed, annuli readings were cross-checked by three independent observers, and ambiguous cases were excluded. The dominance of young individuals (69.4% aged 0+–1+) in our samples suggests that scale-based analysis remains reliable for characterizing this rapidly expanding population. Nevertheless, future studies should prioritize integrating complementary methods such as otolith microstructural analysis or length–frequency modeling, particularly for older age groups, to refine growth parameter estimates and validate the observed age structure.

Age structure and composition are fundamental indicators of population dynamics. In Lugu Lake, the simple age structure (five age groups) and dominance of juveniles suggest minimal fishing pressure [23,28] and rapid population growth. This contrasts with other water bodies like Dali Lake, where *C. auratus* populations exhibit up to 18 age groups under sustained fishing pressure [30,31,32]. The lack of older individuals in Lugu Lake may reflect either recent invasion dynamics or density-dependent growth suppression, both warranting further investigation.

### 4.2. Growth Characteristics of C. auratus in Lugu Lake

The growth of fish is influenced by both genetic and environmental factors. Different fish species typically exhibit distinct growth patterns. Even within the same species, variations in habitat, food availability, and fishing pressure can lead to significant differences in growth and other life history traits [33,34]. By comparing the growth coefficient, growth ratio, inflection point age, asymptotic body length, and asymptotic body weight of *C. auratus* in Lugu Lake with those in other waters, it was observed that these growth parameters varied (Table 3). The growth coefficients of different *C. auratus* populations ranged from 0.0720 to 0.2640, with the population in Lugu Lake exhibiting the lowest growth coefficient and the population in Wanghu Lake showing the highest. The isometric growth (b ≈ 3) observed here aligns with patterns reported in fragmented riverine systems [35] and Mediterranean littoral fishes [36], suggesting environmental stressors may constrain allometric shifts despite invasion advantages. Additionally, the asymptotic body length of *C. auratus* in Lugu Lake was greater than that in other water bodies, while the asymptotic body weight was comparable to that in Caohai Lake but smaller than in other locations. Overall, the growth performance of *C. auratus* in Lugu Lake was inferior to that in other water bodies. This discrepancy may be attributed to differences in genetic resources, geographical location, and food availability among populations. Lugu Lake, situated in a plateau region, has a low average water temperature and relatively scarce fish food organisms, resulting in a generally slow growth rate for the fish.

### 4.3. The Impact of C. auratus Invasion in Lugu Lake and Recommendations for Population Management

The original fish fauna of Lugu Lake is quite simple. In 1978, Kunming Institute of Zoology, Chinese Academy of Sciences, conducted the first investigation of the fishery resources in Lugu Lake, during which they discovered and named three new species of *Schizothorax* fish [41]. However, these *Schizothorax* fish exhibit slow growth rates, making their populations particularly vulnerable to depletion from overfishing.

*C. auratus* was introduced into Lugu Lake through the economic introduction of fish species. In the 1980s, in response to a significant decline in the production of native fish species in Lugu Lake and the unstoppable trend, fishery experts proposed new development strategies. These strategies included replacing the native economic fish species with foreign economic fish species, which involved the introduction of various economic fish and an increase in the number of fry. *C. auratus* was one of the economic fish species introduced during this period. Additionally, local release activities further heightened the likelihood of *C. auratus* invading Lugu Lake. Over time, *C. auratus* gradually reproduced in the lake and became the dominant species. A survey of fish resources in the Lugu Lake Nature Reserve conducted in 2015 revealed that the proportion of *C. auratus* had risen to an astonishing 86%, establishing it as the absolute dominant species [42,43]. This dominance threatens the survival of endemic *Schizothorax* species (<0.5% abundance) [3], primarily through interspecific competition for benthic food resources, predation pressure, and habitat overlap. The adaptability of *C. auratus*, including its rapid acclimatization and stable population dynamics [44], may disrupt the lake’s native trophic web and reduce biodiversity.

Building on *C. auratus’* unique adaptability in nutrient-limited environments [45]. In order to mitigate the impact of the *C. auratus* invasion on the ecosystem of Lugu Lake, the following three measures are recommended. First, it is essential to conduct irregular, centralized fishing and removal efforts in areas of the lake where *C. auratus* are densely occurring. Second, increasing the intensity of artificial breeding and propagation of indigenous fish is crucial to rapidly expand their population size. Third, enhancing the public’s awareness and understanding of invasive species through education and outreach initiatives will help reduce human activities that contribute to the problem, such as the release of non-native species [42].

## 5. Conclusions

This study reveals that the invasive *C. auratus* population in Lugu Lake exhibits a simplified age structure (0+–4+ years), with juveniles (69.4%) predominating, characteristic of *r*-selected species expansion. The weight–length relationship (*W* = 2 × 10^−5^*L*^3.026^) indicates isometric growth, yet the lower growth coefficient (*k* = 0.0721) compared to low-altitude populations suggests environmental suppression. Contrasting with aquaculture studies [30], wild populations adapt to oligotrophic conditions through delayed growth inflection points (6.66 years) and optimized energy allocation [43]. We recommend integrated management combining targeted fishing during reproduction and plankton biomass control, providing a paradigm for invasive fish control in plateau lakes.

## Figures and Tables

**Table 1:** Seasonal averages of water quality parameters at Lugu Lake sampling sites.

Sampling Site	Temp (°C)	pH	DO (mg/L)	Conductivity (μS/cm)	TN (mg/L)	TP (mg/L)	TSS (mg/L)	COD (mg/L)
Dazu	17.43	8.27	7.19	238.5	1.1	0.34	1.9	24.75
Xiaoluoshui	17.03	8.43	7.24	239.8	1.08	0.19	1.5	15.75
Xiaoyuba	17.33	8.37	7.15	238.5	0.6	0.19	1.75	33.75
Nvshenwan	17.03	8.27	7.21	239.8	0.63	0.12	1.5	23.75
Langfang	18.35	8.28	7.16	238.8	1.5	0.14	1.25	45.25
Caohai	17.15	8.4	7.31	236.3	1.2	0.26	2.5	3.88
Haimen	16.8	7.19	3.47	247.5	1.08	0.24	3	30.5

**Table 2:** Back-calculated body length of different age groups of *C. auratus* Lugu Lake.

Age Group	Calculate the Body Length/mm				Sample Size
	L1	L2	L3	L4	
0+					347
1+	67.41				118
2+	67.91	112.25			82
3+	66.43	110.77	147.22		89
4+	63.47	104.86	141.80	173.82	34
Average	66.31	109.29	144.51	173.82	670

**Table 3:** Comparison of growth parameters of *C. auratus* in different water areas.

Location	Lake Network	Lake Baikal	South Bay Reservoir	Poyang Lake	Caohai Lake	Lugu Lake
Growth coefficient	0.2640	0.1797	0.1990	0.1424	0.1537	0.0720
Growth rate	2.9274	2.8179	4.0060	2.8871	2.9024	2.9113
Inflection point age	3.95	5.60	7.90	7.13	6.28	6.66
Asymptotic body, length/mm	285.82	301.00	301.20	294.90	259.67	401.57
Asymptotic body, mass/g	653.30	832.30	1097.32	776.96	481.94	484.38
References	[37]	[30]	[38]	[39]	[40]	This study

## Data Availability

Data are contained within the article and will be available upon request.
